# Antimicrobial Potential of Probiotic Strains From Bulgarian Cheese and Shallot Yogurt Against *Staphylococcus saprophyticus*

**DOI:** 10.1155/2024/2978083

**Published:** 2024-10-15

**Authors:** Pardis Amigh, Yasaman Ahmadi, Milad Mohkam, Dariush Shokri

**Affiliations:** ^1^Department of Biotechnology, Faculty of Biological Sciences and Technology, Shahid Ashrafi Esfahani University, Isfahan, Iran; ^2^Department of Microbiology, Kish International Branch of Islamic Azad University, Kish, Iran; ^3^Allergy Research Center, Shiraz University of Medical Sciences, Shiraz, Iran; ^4^Nosocomial Infection Research Center, Isfahan University of Medical Sciences, Isfahan, Iran

**Keywords:** antibiofilm, antimicrobial activity, *Lactobacillus rhamnosus*, probiotics, *Staphylococcus saprophyticus*

## Abstract

The escalating incidence of hospital infections due to antibiotic resistance necessitates the identification of alternative therapeutic agents such as probiotics. This study was designed to isolate and evaluate the efficacy of probiotics against *Staphylococcus saprophyticus*, a prevalent etiological agent of urinary tract infections (UTIs). A total of 100 *S. saprophyticus* strains were isolated from clinical samples and subjected to antibiotic susceptibility testing via the disc diffusion method. Concurrently, probiotic bacteria were isolated from Bulgarian cheese and shallot yogurt, and their antibacterial activity against *S. saprophyticus* strains was assessed. The inhibitory potential of probiotic supernatants was evaluated using microtiter plate assays, with the minimum inhibitory concentration (MIC) and minimum bactericidal concentration (MBC) determined at a 1/2 dilution. Cytotoxicity was evaluated using the MTT assay, and high-performance liquid chromatography (HPLC) was employed to analyze the concentrations of organic acids produced by the probiotics. The results revealed that all *S. saprophyticus* strains were resistant to tetracycline and doxycycline but susceptible to other antibiotics. *Lactobacillus rhamnosus* strains M and B demonstrated notable antibacterial and antibiofilm activity against *S. saprophyticus* isolates. These probiotics exhibited susceptibility to most antibiotics and lacked virulence factors, suggesting their safety for therapeutic use. The organic acids produced by the probiotics were identified as lactic acid, acetic acid, and formic acid. In conclusion, *L. rhamnosus* strains M and B exhibit potent antimicrobial properties against *S. saprophyticus*, indicating their potential as therapeutic agents for UTIs. Further research is warranted to validate these findings and explore the possibility of these probiotics in clinical applications.

## 1. Introduction

An uncomplicated urinary tract infection (UTI) refers to a bacterial infection that specifically affects the bladder and related anatomical tissues. This condition is observed in people who do not exhibit any structural abnormalities or concurrent medical conditions, such as diabetes, immunodeficiency, or pregnancy. Uncomplicated UTI is alternatively referred to as cystitis or lower UT [1]. *Staphylococcus saprophyticus*, a gram-positive, coagulase-negative coccus, is identified as a uniquely causative agent of uncomplicated UTIs. It is estimated to be responsible for approximately 10%–20% of UTIs acquired within the community, primarily affecting sexually active females in their younger age groups [2, 3]. *S. saprophyticus* can stick to uroepithelial cells and form biofilms, which play a significant role in its pathogenicity and antibiotic resistance [4]. The prevalence of uncomplicated UTIs caused by *S. saprophyticus* is impacted by various factors, including sexual activity, contraceptive usage, genetic predisposition, and anomalies in the urinary system [4, 5]. The adhesion and biofilm formation of *S. saprophyticus* is facilitated by a range of molecular processes and factors, including surface proteins, extracellular polysaccharides, quorum sensing, and environmental conditions [6, 7]. The current treatment approach for UTIs caused by *S. saprophyticus* encounters various problems and constraints, including rising rates of resistance, side effects, recurrence, and ecological consequences [8]. Hence, it is imperative to explore alternate approaches for the prevention and treatment of uncomplicated UTIs caused by *S. saprophyticus*.

One potential strategy is the utilization of probiotics, which are live bacteria that provide advantageous effects on the host's health when provided in sufficient quantities [9]. Probiotics can demonstrate antimicrobial properties against a range of pathogens, such as *S. saprophyticus*, through the production of organic acids, hydrogen peroxide, bacteriocins, and other bioactive compounds [10, 11]. In addition to their known effects, probiotics can regulate the immune system of the host, improve the function of the intestinal barrier, and prevent the attachment and infiltration of harmful microorganisms [12].

Probiotics are present in a range of fermented dairy products, including yogurt and cheese, which have a long-standing cultural consumption history in numerous nations [13]. Bulgarian cheese and shallot yogurt are notable for their abundance of probiotic bacteria from the *Lactobacillus* and *Bifidobacterium* genera, which have demonstrated advantageous impacts on human well-being. The aforementioned effects encompass the enhancement of the gut microbiota, fortification of the immune system, prevention of infections, reduction of cholesterol and blood pressure levels, and regulation of inflammation [14, 15]. Bulgarian yogurt and cheese are produced by employing distinct starter cultures comprising strains of *Lactobacillus delbrueckii* subsp. bulgaricus and *Streptococcus thermophilus*, alongside other indigenous microorganisms, play a role in shaping the sensory and functional characteristics of these food items [16]. The starter cultures possess unique molecular and biochemical attributes, including proteolytic, lipolytic, and glycolytic activities, as well as the ability to produce organic acids, volatile compounds, and exopolysaccharides. Additionally, they exhibit interactions with other microorganisms [17].

Shallot yogurt is a traditional fermented dairy product that enjoys popularity in Bulgaria and other countries within the Balkan region. The production process involves the incorporation of finely diced shallots (*Allium cepa* var. aggregatum) into milk, which is subsequently fermented using yogurt starter culture. The unique taste and scent of shallot yogurt can be attributed to the sulfur compounds found in shallots, which are also known to possess antibacterial and antioxidant properties [18]. Shallot yogurt exhibits commendable nutritional and sensory characteristics, including its notable levels of protein, calcium, phosphorus, vitamin C, and dietary fiber, as well as its minimal quantities of lactose and fat. Additionally, it is distinguished by its appealing taste, texture, and color [18, 19].

The objective of this research is to assess the antibacterial efficacy of probiotic microorganisms derived from Bulgarian cheese and shallot yogurt against *S. saprophyticus*. It is postulated that these strains possess the capability to impede the proliferation and production of biofilms in *S. saprophyticus* by the synthesis of diverse antimicrobial agents and by vying for adhesion sites. Additionally, we explore the potential mechanisms of action associated with these probiotic strains, as well as their safety and compatibility with human cells.

## 2. Materials and Methods

### 2.1. Isolation of *Lactobacillus* Strains

In total, six samples of fresh Bulgarian cheese and shallot yogurt were randomly collected from local markets in Isfahan, Iran, and designated as A1 to A6. All samples were collected and processed on the same day. Following collection, the samples were promptly transported to the laboratory under refrigeration conditions (4°C) to prevent spoilage. Subsequently, 2 g of each sample were aseptically dissolved in 18 mL of normal saline (at a 1:10 *w*/*v* ratio) and agitated at 600 rpm for 10 min. Thereafter, 1 mL of each prepared sample was aseptically transferred to 10 mL of MRS broth media (Merck, Germany) within a microaerobic environment enriched with 5% CO_2_. These sample dilutions (0.01 mL) were then plated onto MRS agar plates (Merck, Germany) and subsequently incubated for 48 h. Following incubation, colonies exhibiting growth on the agar plates were subcultured into a broth culture medium and further incubated at 37°C for 24 h. For subsequent analysis, the isolated bacterial strains were preserved by submerging them in a preservation medium consisting of 10% skim milk (*w*/*v*) and 30% glycerol (*w*/*v*) and maintained at −70°C [20].

### 2.2. Isolation and Identification of Clinical *S. saprophyticus* spp.

Urine samples were collected from subjects on clinical signs of UTI diagnosed by licensed health care professionals based on reported symptoms and clinical evaluation. Clean-catch midstream urine samples were collected in sterile containers using standard aseptic techniques. Patient confidentiality was maintained by using sample codes that deidentified the samples. Afterward, the samples were subjected to cultivation on blood agar and MacConkey agar media. The plates were subjected to incubation at a temperature of 37°C for a duration of 24 h, after which they were assessed for the presence of bacterial proliferation. The gram-positive cocci, which exhibited catalase-positive and coagulase-negative characteristics, were subsequently subjected to a novobiocin susceptibility test for further identification. A circular medium infused with 5 *μ*g of novobiocin was positioned on a bacterial growth layer spread on Mueller–Hinton agar. The agar plate was then subjected to incubation at a temperature of 37°C for a duration of 18 h. The measurement and interpretation of the zone of inhibition surrounding the disc were conducted following the recommendations established by the Clinical and Laboratory Standards Institute (CLSI) [21]. Novobiocin-resistant isolates with a zone diameter of ≤ 16 mm were identified as *S. saprophyticus.*

### 2.3. Antibiotic Susceptibility Evaluation

The antibiotic resistance patterns of clinical isolates of *S. saprophyticus* were assessed by employing the disc diffusion technique following the recommendations set by the CLSI [21]. The antibiotics that were utilized were listed as follows: cefoxitin (30 *μ*g), erythromycin (15 *μ*g), clindamycin (2 *μ*g), gentamycin (120 *μ*g), doxycycline (30 *μ*g), nitrofurantoin (300 *μ*g), linezolid (30 *μ*g), cefoperazone (75 *μ*g), rifampicin (5 *μ*g), trimethoprim/sulfamethoxazole (1.25/23.75 *μ*g), and tetracycline (30 *μ*g). Moreover, the following antibiotics were used to test the susceptibility of probiotic isolates: nitrofurantoin (300 *μ*g), erythromycin (15 *μ*g), clindamycin (2 *μ*g), gentamycin (120 *μ*g), ciprofloxacin (5 *μ*g), ampicillin (10 *μ*g), linezolid (30 *μ*g), penicillin (10 *μ*g), rifampicin (5 *μ*g), tetracycline (30 *μ*g), trimethoprim/sulfamethoxazole (1.25/23.75 *μ*g), and vancomycin (30 *μ*g).

### 2.4. Agar Well Diffusion Method

The Muller–Hinton agar plates were prepared and inoculated with 0.1 mL of a suspension of *S. saprophyticus* containing 10^6^ colony-forming units per milliliter. The suspension was evenly spread across the entire surface of the agar plates. Afterward, sterile cork borers were used to punch 6 mm-diameter holes into the agar. Next, 50 *μ*L of Bulgarian cheese and shallot yogurt extracts were added to their corresponding wells using a micropipette. The plates were incubated at 37°C for 24 h to promote the diffusion of antimicrobial agents. After incubation, the diameter of the zones of inhibition surrounding each well was measured using a ruler, and the measurements were recorded. The assay was performed in triplicate to evaluate the antimicrobial efficacy of the extracts or supernatants against the growth of *S. saprophyticus* [22].

### 2.5. Time–Kill Test in Cocultures

This experiment is aimed at identifying the most effective probiotic strain within a specified timeframe. The time–kill assay involved combining *S. saprophyticus* cells with the cell-free supernatant of *Lactobacillus* spp. for coculture. A suspension with a turbidity equivalent to 0.5 McFarland was prepared using the *S. saprophyticus* strain. Next, the supernatant was added to the suspension. The sample was cultured on blood agar medium and incubated at 37°C for 24 h at various time points: 0, 1, 2, 4, 8, 12, 24, and 48 h [22].

### 2.6. Cold Enrichment of Probiotic Bacteria

BHI broth was prepared and cooled to a low temperature (4°C) to create a cold environment. Probiotic cultures were inoculated into the chilled BHI broth, and the mixture was incubated at the specified low temperature (4°C) for 24 h. This cold enrichment process allowed for the selective proliferation of probiotic bacteria while suppressing the growth of nonprobiotic or pathogenic microorganisms. Subsequently, bacterial growth in the medium was assessed by observing turbidity [22].

### 2.7. Broth Microdilution Method

The measurement of antibacterial activity, namely, the minimum inhibitory concentration (MIC) and minimum bactericidal concentration (MBC), was conducted on cell-free supernatants derived from probiotics that were cultivated in the presence of clinical isolates of *S. saprophyticus*. No further turbidity was observed thereafter to ascertain accuracy. The pathogenic strain was subsequently cultivated on a blood agar medium and subjected to an additional incubation period of 24 h at 37°C. This investigative process revealed the inhibitory effect of probiotics on the pathogenic strain, shedding light on their potential as agents against *S. saprophyticus* [22].

### 2.8. Antibiofilm Effect of Lactobacilli

A 24-well plate was utilized to assess the antibiofilm activity of various strains of lactobacilli isolates against the pathogenic bacterium *S. saprophyticus*. Lactobacilli supernatants were acquired after an overnight culture at 37°C in MRS broth. To isolate the bacterial cells from the supernatant, the cultures were centrifuged at 10,000 × g for 15 min. Filtration was performed on the supernatants using a 0.22 *μ*m filter in order to remove any remaining bacterial cells. Until further use, the sterile supernatants were stored at −80°C. The evaluation of *S. saprophyticus* biofilm formation was conducted utilizing 24-well tissue culture plates. After dilution of overnight cultures of *S. saprophyticus* to an optical density at 600 nm (OD600) of 0.1, each well received 1 mL of this bacterial suspension. For the formation of biofilm, plates were incubated at 37°C for a duration of 24 h. To evaluate the antibiofilm activity of supernatants derived from lactobacilli, different dilutions of the supernatants were introduced into *S. saprophyticus* biofilms. Plates were incubated for an additional 24 h. Without supernatant treatment, *S. saprophyticus* biofilms were present in the control wells. The biofilms were quantified using crystal violet staining, and the optical density was determined at 570 nm following incubation. Experiments were conducted in triplicate, and significant differences were determined through statistical analysis [23].

### 2.9. High-Performance Liquid Chromatography (HPLC) of Lactobacilli Supernatants

*Lactobacillus* isolates were grown in MRS broth for 72 h. The cultures were then centrifuged at 10,000 g for 10 min. The supernatant was carefully collected and filtered through a 0.22 *μ*m syringe to ensure sterility. To ensure lactobacilli-free growth, the filtrate was recultivated in MRS broth medium for 72 h. The HPLC apparatus received 20 *μ*L of the sterile filtrate after sterility verification. The separation of chemicals was done using reversed-phase HPLC columns C18 (25 cm, 4.6 mm). An aqueous solution of phosphate buffer and acetonitrile (CH_3_CN) at 10 mM and pH 3.6 was used as the mobile phase. To identify and quantify the chemical, UV absorbance at 282 nm was measured at room temperature, 25°C, after the separation at 1 mL/min. A thorough HPLC methodology was used to analyze lactobacilli supernatants, ensuring reliable and reproducible results [22].

### 2.10. Acid and Bile Salt Tolerance Assay

The acid and bile salt tolerance of lactobacilli isolates were assessed using the methodology described by Duc et al. [24], with minor modifications. In this study, an MRS broth with a pH of 2.5 was created and subsequently infected with a bacterial cell suspension including all of the isolates. Samples of 0.5 mL were examined at 0, 1, 2, and 3 h to determine the total number of live cells. The plate counts on MRS agar were subjected to incubation at a temperature of 37°C for a duration of 24 h under a pH condition of 2.5. To assess bile salt tolerance, an MRS broth supplemented with oxgall (0.3%) was prepared and afterwards inoculated with 1 mL of a 48-h-old culture. Samples were collected at 0- and 3-h intervals. The dilutions that were deemed suitable were directly plated onto nutrient agar plates. The plates were subjected to incubation at a temperature of 37°C for a duration of 24 h to ascertain the number of colony forming units (CFU). After experimenting three times, the mean values of each bacterial isolate were considered [20].

### 2.11. Cytotoxicity Assay

The 3-(4,5-dimethylthiazol-2-yl)-2,5-diphenyltetrazolium bromide (MTT) assay was employed to evaluate the cytotoxicity of lactobacilli supernatants on L929 fibroblast cells. The assay employs MTT as a measure of cell viability. The L929 cells were cultured in Dulbecco's Modified Eagle Medium (DMEM) supplemented with 10% fetal bovine serum (FBS) and 1% penicillin–streptomycin. The cells were maintained at a temperature of 37°C with 5% CO_2_ until they reached 80% confluence. Different dilutions of lactobacilli supernatants were introduced to L929 cells, which were subsequently incubated for 24 h. The control wells consisted of cells that were cultured in a medium without any treatment of the supernatant. Following the incubation period, the addition of MTT solution was carried out for each well, and subsequent solubilization of formazan crystals was achieved using dimethyl sulfoxide (DMSO). Cell viability as a percentage of the control was determined by measuring the absorbance at 570 nm using a microplate reader (BioTek Instruments Inc., Vermont, United States). The cytotoxicity assessment was conducted in triplicate to ensure experimental reliability [9].

### 2.12. Identification of Lactobacilli Isolates

A multistep procedure was required to identify potent *Lactobacillus* isolates that exhibited inhibitory and antibiofilm properties against pathogenic strains. In accordance with established protocols [20], isolates were initially chosen based on their classical characteristics, which included morphology, cultural traits, and biochemical attributes. The strains that were initially identified using conventional methods were subsequently verified through 16S ribosomal DNA (rDNA) PCR using universal primers: Universal 2 (1492r): TACGGYTACCTTGTTACGACTT and Universal 1 (27f): AGAGTTTGATCCTGGCTCAG (Cinnagen Company, Iran), respectively. To amplify the 16S rDNA gene, the amplification protocol consisted of the subsequent steps: an initial denaturation cycle at 94°C for a duration of 5 min; 30 cycles of denaturation at 94°C for 30 s, annealing at 54°C for 30 s, and extension at 72°C for 5 min; and a final extension cycle at 72°C for 5 min. Following this, the positive PCR products underwent nucleotide sequencing at the facility of Macrogen Inc. (Seoul, Korea). Following this, the acquired sequences underwent BLAST searches in the database of the National Center for Biotechnology Information (NCBI) to ensure their accuracy and registration.

### 2.13. Statistical Analysis

Each experiment was performed three times, and the mean and standard error values were calculated using SPSS software (SPSS Inc. No. 22).

## 3. Results

### 3.1. *S. saprophyticus* Isolation and Antibiotic Sensitivity Pattern

The isolation and identification of 100 S*. saprophyticus* strains from clinical samples were performed by morphological and biochemical tests. The most common source of *S. saprophyticus* was the cervix (45%), followed by urine (35%), urethra (15%), and other sites (5%), respectively. The antibiotic susceptibility pattern of clinical *S. saprophyticus* isolates revealed that all strains (100%) were resistant to tetracycline and doxycycline, which are commonly used antibiotics for UTIs. However, all strains (100%) were susceptible to the other antibiotics tested, such as clindamycin, gentamycin, nitrofurantoin, and linezolid (Table 1).

### 3.2. Isolation, Screening, and Identification of Lactobacilli

Based on gram staining, catalase test, and cell morphology, 33 out of 67 isolates from Bulgarian cheese and shallot yogurt were classified as lactic acid bacteria (LAB). All isolates were gram-positive, catalase-negative, and *γ*-hemolytic. The isolates exhibited different morphologies, such as cocci in pairs or chains and rods in pairs or chains. Two isolates, named M and B, showed the highest antibacterial activity against clinical *S. saprophyticus* isolates, which were isolated from shallot yogurt and Bulgarian cheese, respectively. Further identification by 16S rRNA gene sequencing revealed that they belonged to the genus *Lactobacillus rhamnosus* M and B with the assigned accession numbers PQ083436 and PQ083437, respectively. Moreover, they demonstrated resistance to bile and acid stress, as well as the ability to grow at low temperature (4°C) for 24 h. Finally, the genome sequences of the two *Lactobacillus* strains, M and B, were deposited in the NCBI database and assigned the accession numbers ON837155.

### 3.3. Antimicrobial Assessment of Lactobacilli Isolates Against *S. saprophyticus*

The inhibitory impact of the supernatants from probiotic bacteria M and B on *S. saprophyticus* was established by the utilization of the 24-well microtiter plate assay. No significant alteration in optical density was detected in any of the wells when the supernatants were cocultivated with *S. saprophyticus*. Following a 24-h incubation period, it was observed that the wells containing 1 and 1/2 dilutions of the supernatants did not exhibit any bacterial growth. This observation suggests that these particular concentrations demonstrated bactericidal properties against *S. saprophyticus*. Nevertheless, it was observed that bacterial colonies were present in the wells containing supernatant dilutions of 1/4, 1/8, and 1/16. This observation suggests that these concentrations were inadequate in inhibiting the growth of *S. saprophyticus*. Therefore, the MIC of the probiotic supernatants against *S. saprophyticus* was determined as 1/2 dilution, based on the absence of turbidity in the 24-well microtiter plate assay.

To determine the MBC, 50 *μ*L of the supernatants from the wells with no turbidity (1 and 1/2 dilution) were transferred to blood agar plates using sterile swabs and incubated at 37°C for 24 h. No bacterial growth was observed on the plate with 1/2 dilution of the supernatants, indicating that this concentration was bactericidal for *S. saprophyticus*. Therefore, the probiotic supernatants had the same concentration for both MBC and MIC against *S. saprophyticus*.

The antibiofilm activity of the probiotic supernatants was also evaluated by measuring the optical density of the biofilms formed by *S. saprophyticus* in the presence of different concentrations of the supernatants. The results showed that the probiotic supernatants inhibited biofilm formation by *S. saprophyticus* at 1, 1/2, and 1/4 dilutions, but not at 1/8 and 1/16 dilutions, suggesting that these concentrations were subinhibitory for *S. saprophyticus*.

### 3.4. Pattern of Antibiotic Susceptibility and Virulence Factor of Probiotics

The antibiotic susceptibility patterns of the probiotic strains M and B were determined by the disc diffusion method. Both strains were sensitive to most of the tested antibiotics, such as erythromycin, clindamycin, gentamycin, ciprofloxacin, ampicillin, linezolid, penicillin, rifampicin, and tetracycline. However, both strains were resistant to trimethoprim/sulfamethoxazole and vancomycin. Moreover, strain M was susceptible to nitrofurantoin, whereas strain B was resistant to it.

The virulence factors of the probiotic strains M and B were assessed by testing their ability to produce DNase, *β*-hemolysin, catalase, CAMP factor, and gelatinase enzymes. Both strains were negative for all these tests, indicating that they lacked these virulence factors.

### 3.5. Cytotoxicity Assay

The cytotoxicity of the probiotic strains M and B was evaluated by measuring their effect on the viability of the L929 fibroblast cell line using the MTT assay. The results showed that the cytotoxicity of strain M was 7.1% and that of strain B was 6.3, which was not significantly different from the cytotoxicity of the control sample of 6.5% (*p* > 0.05). This indicated that both probiotic strains had a low level of cytotoxicity and did not adversely affect the viability of the L929 fibroblast cell line.

### 3.6. HPLC Analyzing

The inhibitory effect of the organic acids produced by the probiotic strains M and B on *S. saprophyticus* was investigated by neutralizing the acidic pH of the culture supernatants. The results showed that all *S. saprophyticus* strains were able to grow in the neutralized supernatants, indicating that the organic acids were responsible for the antibacterial activity of the probiotics.

**Figure 1:**
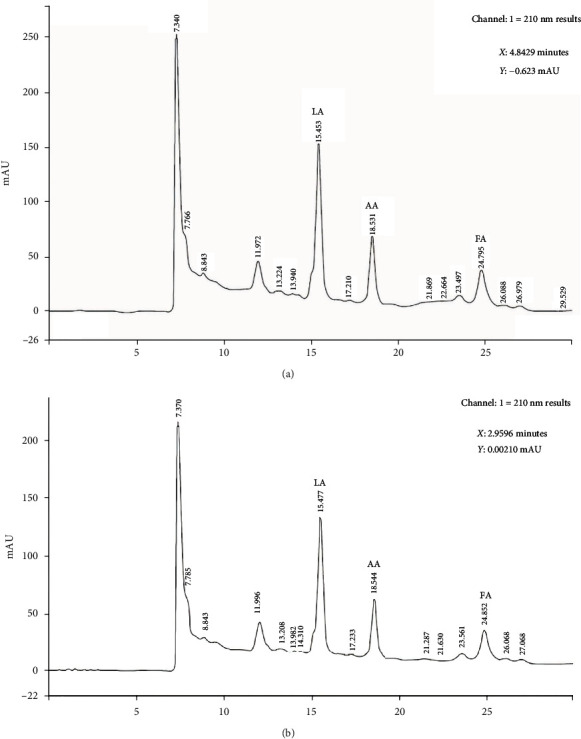
HPLC graphs of the analysis of the type and amount of organic acids in two *Lactobacillus* (a) M and (b) B strains. LA, lactic acid; AA, acetic acid; FA, formic acid.

The type and concentration of the organic acids were determined by HPLC analysis. The results revealed that the probiotic strains M and B produced three organic acids: formic acid, lactic acid, and acetic acid. The concentration of each organic acid varied between the two strains. Strain M produced more acetic acid (535.2 mg/L) than formic acid (280.8 mg/L) and lactic acid (254.3 mg/L). Strain B produced more formic acid (338.4 mg/L) and acetic acid (301.4 mg/L) than lactic acid (174.7 mg/L) (Figure 1).

## 4. Discussion

UTIs, particularly those caused by *S. saprophyticus*, a gram-positive coccus found in the normal flora of the female genital tract and perineum, present significant challenges due to their prevalence and the emergence of antibiotic resistance [1, 2, 25]. The bacterium's ability to adhere to uroepithelial cells and persistently grow in the urinary tract makes it a common cause of community-acquired UTIs [2, 26]. This study is aimed at exploring the potential of probiotic strains, such as *L. rhamnosus*, derived from Bulgarian cheese and shallot yogurt as alternatives for combating *S. saprophyticus* infections. *L. rhamnosus*, originally considered a subspecies of *L. casei* but later identified as a separate species through genetic research, is a gut-friendly bacterium used as a probiotic, particularly useful in treating infections of the female urogenital tract [27, 28]. It can help relieve diarrhea, IBS symptoms, and cavities, and aid gut health, teeth, and oral health. *Lactobacillus rhamnosus*, adept at thriving within the gastrointestinal tract, presents promising long-term advantages [29]. It is commonly employed as a probiotic supplement and frequently incorporated into various dairy products such as yogurts, cheeses, and milk to augment their probiotic constituents [30]. The findings provide insights into the antimicrobial efficacy, safety, and mechanisms of action of these probiotic strains, offering a better understanding of both *L. rhamnosus* and *S. saprophyticus*, the latter being part of the normal flora in humans that colonizes areas like the perineum, rectum, urethra, cervix, and gastrointestinal tract, despite being a common cause of UTIs [2]. This understanding can help in developing effective strategies for the prevention and treatment of UTIs.

Antibiotic susceptibility of *S. saprophyticus* plays a critical role in managing UTIs, particularly in young, sexually active women [2, 31]. The effectiveness of treatment regimens directly depends on the bacterium's resistance or sensitivity to antibiotics. In UTIs, antibiotic-resistant *S. saprophyticus* strains can lead to treatment failure, prolonged infection, and increased complications [32]. For instance, resistance to tetracycline and doxycycline is common, necessitating the use of alternative antibiotics such as clindamycin, gentamycin, nitrofurantoin, and linezolid [33]. Our study's findings align with a broader understanding, showing complete resistance to tetracycline and doxycycline but susceptibility to other tested antibiotics [33, 34]. Despite specific resistance mechanisms, *S. saprophyticus* remains susceptible to various antimicrobial agents, emphasizing the importance of appropriate antibiotic selection based on susceptibility patterns. Moreover, recent research elucidates *S. saprophyticus*'s antibiotic resistance further. Studies reveal multidrug resistance and genetic markers like the mecA gene associated with resistance to cefoxitin and oxacillin [35, 36]. Biofilm formation also contributes to resistance against several antibiotics [37, 38]. However, our study did not find resistance to erythromycin and clindamycin, contrasting previous findings, possibly due to variations in bacterial strains or experimental conditions.

The antimicrobial assessment of lactobacilli isolates against *S. saprophyticus* in UTIs is a critical area of research, especially in the context of increasing antibiotic resistance. Lactobacilli, a genus of gram-positive bacteria commonly found in the human microbiota, have been studied for their potential to inhibit the growth of uropathogens, including *S. saprophyticus*, which is responsible for a significant proportion of UTIs [5, 11]. Our findings contribute to this body of research by demonstrating that specific lactobacilli isolates from Bulgarian cheese and shallot yogurt exhibit antibacterial activity against *S. saprophyticus*. This activity is particularly notable in the inhibition of biofilm formation, a key factor in the persistence and recurrence of UTIs [5]. Biofilms are complex structures that bacteria like *S. saprophyticus* form on surfaces, such as the epithelial lining of the urinary tract, which protect them from antibiotics and the host immune response [5]. The lactobacilli isolates' ability to produce organic acids, such as lactic acid, acetic acid, and formic acid, is believed to be one of the mechanisms behind their antimicrobial properties. These acids can lower the pH of the environment, making it inhospitable for the growth of pathogenic bacteria. Additionally, lactobacilli can compete with pathogens for adhesion sites on the host epithelium, further preventing infection [12]. In this regard, our study aligns with recent research that emphasizes the need for novel treatments against UTIs due to the rising rates of antibiotic resistance [39, 40]. The specificity of the probiotic strains' activity against *S. saprophyticus* presents a compelling case for their use as an alternative to traditional antibiotics, which are becoming increasingly ineffective [11, 41]. The antimicrobial assessment in our study showed that the supernatants from lactobacilli strains M and B were effective in inhibiting the growth of *S. saprophyticus*, with the MIC determined to be at a 1/2 dilution. This suggests that substances secreted by these probiotic strains have the potential to be used as novel agents in the treatment of UTIs caused by *S. saprophyticus* [42].

Biofilms play a significant role in UTIs by providing a protective environment for bacteria, which can lead to persistent infections and complicate treatment. Biofilms are complex communities of bacteria that adhere to surfaces and are encased in a self-produced matrix of carbohydrates, proteins, fats, and DNA [43]. This matrix shields the bacteria from the immune system and antibiotics, making infections difficult to eradicate and often leading to recurrent UTIs [43]. In the context of our study, the *L. rhamnosus* strains M and B demonstrated the ability to inhibit the growth and biofilm production of *S. saprophyticus*. This is particularly relevant because biofilms contribute to the pathogenicity of UTIs and are associated with antibiotic resistance. By preventing biofilm formation, these probiotic strains could potentially reduce the incidence of recurrent UTIs and offer an alternative to traditional antibiotic treatments, which are less effective against biofilm-embedded bacteria [44]. Furthermore, probiotics themselves can form biofilms on host mucosal surfaces, which may competitively exclude pathogenic bacteria, preventing their colonization and subsequent infection [45]. This suggests that the introduction of beneficial probiotic bacteria could help in establishing a protective biofilm that outcompetes harmful pathogens, thereby reducing the risk of UTIs.

The pattern of antibiotic susceptibility and the presence of virulence factors in probiotics are critical considerations when evaluating their potential use in treating UTIs [46]. In our study, the probiotic strains M and B showed a broad spectrum of antibiotic susceptibility, being sensitive to most of the tested antibiotics, which is a desirable trait for probiotics used in a clinical setting [47]. This suggests that these strains can be safely administered alongside conventional antibiotics without the risk of contributing to antibiotic resistance [48]. Moreover, the absence of virulence factors in the probiotic strains M and B, as indicated by negative results for cell cytotoxicity, DNase, *β*-hemolysin, catalase, CAMP factor, and gelatinase, is significant. Probiotics intended for therapeutic use should not possess virulence factors that could potentially harm the host [49]. The lack of these factors in strains M and B supports their safety profile and suitability for use in managing UTIs [48]. The relationship between antibiotic susceptibility, virulence factors, and UTI treatment outcomes is complex. Uropathogens with multiple virulence factors are often more challenging to treat and may exhibit higher levels of antibiotic resistance [50]. Conversely, probiotics that are susceptible to antibiotics and lack virulence factors can be advantageous in UTI treatment. They can provide a competitive edge against uropathogens by occupying niches in the urinary tract, thereby preventing infection, and can be used in conjunction with antibiotics to enhance treatment efficacy [51, 52].

In conclusion, this study explored the potential of lactobacilli strains, particularly *L. rhamnosus* from Bulgarian cheese and shallot yogurt, as an alternative approach for the treatment of UTIs caused by *S. saprophyticus*. The findings demonstrate that these probiotic strains exhibit antibacterial activity against *S. saprophyticus*, including inhibiting biofilm formation, a crucial factor in UTI persistence and recurrence. Moreover, exploring the synergistic effects of combining these probiotics with antibiotics could open new therapeutic modalities. The development of functional foods or supplements containing these strains could also provide a practical means of preventing UTIs, contributing to the overall well-being of individuals at risk. The proposed mechanism involves organic acid production by lactobacilli, creating an inhospitable environment for *S. saprophyticus*. Furthermore, these probiotic strains displayed a broad spectrum of antibiotic susceptibility and lacked virulence factors, highlighting their safety for potential clinical applications. Future research should focus on clinical trials to validate the efficacy and safety of these probiotic strains in human subjects. Overall, this study provides valuable insights into the use of lactobacilli strains as a promising strategy for UTI prevention and treatment, particularly in the face of increasing antibiotic resistance in *S. saprophyticus*.

## Figures and Tables

**Table 1:** The antibiotic resistance patterns of *S. saprophyticus* isolates.

	**FOX**	**E**	**CC**	**GM**	**D**	**FM**	**LZ**	**CP**	**RA**	**SXT**	**TE**
Resistant (%)	—	—	—	—	100	—	—	—	—	—	100
Sensitive (%)	100	100	100	100	—	100	100	100	100	100	—

## Data Availability

Data are available on request from the authors.
